# Utility of Real-Time and Retrospective Continuous Glucose Monitoring in Patients with Type 2 Diabetes Mellitus: A Meta-Analysis of Randomized Controlled Trials

**DOI:** 10.1155/2019/4684815

**Published:** 2019-01-15

**Authors:** Satoshi Ida, Ryutaro Kaneko, Kazuya Murata

**Affiliations:** Department of Diabetes and Metabolism, Ise Red Cross Hospital, Mie, Japan

## Abstract

In the present study, we aimed to investigate the effects of continuous glucose monitoring (CGM) on blood glucose levels, body weight, blood pressure, and hypoglycaemia in patients with type 2 diabetes mellitus using a meta-analysis of randomized controlled trials (RCTs). A literature search was performed using MEDLINE, Cochrane Controlled Trials Registry, and ClinicalTrials.gov. RCTs using CGM in patients with type 2 diabetes mellitus were then selected. Statistical analysis included calculation of the standardized mean difference (SMD) or risk ratio and 95% confidence intervals (CIs) using a random effects model. After literature search, seven RCTs (669 patients) satisfied the eligibility criteria established herein and were included into the meta-analysis. Compared with the self-monitoring blood glucose group, the CGM group exhibited significantly lower HbA1c levels (SMD, −0.35; 95% CI, −0.59–−0.10; *P* = 0.006) and shorter time spent with hypoglycaemia (SMD, −0.42; 95% CI, −0.70–−0.13; *P* = 0.004). Conversely, no differences in body weight and blood pressure were observed between the groups. CGM in patients with type 2 diabetes mellitus could reduce HbA1c levels and time spent with hypoglycaemia. However, because few RCTs were included in this present study and heterogeneity was also noted, care should be taken when interpreting the results.

## 1. Introduction

The number of patients suffering from type 2 diabetes mellitus is increasing worldwide, with estimates suggesting that approximately 300 million individuals could develop the disease by 2050 [1, 2]. Previous studies have revealed that strict blood glucose control is extremely important for preventing microangiopathy and macrovascular disorders [3, 4]. Primary treatment for type 2 diabetes mellitus includes diet/exercise therapy, whereas pharmacotherapy is administered only when diet therapy/exercise therapy is insufficient. However, in many cases, favourable blood glucose control cannot be achieved through the aforementioned therapeutic interventions alone [5, 6].

Self-monitoring blood glucose (SMBG) has been proven to be useful for long-term glycaemic control in patients with type 2 diabetes mellitus [7]. However, this method places considerable burden on the patient given that performing finger pricking several times per day is not only troublesome but also painful [8]. Furthermore, understanding detailed blood sugar fluctuations, such as elevated blood glucose after meals or asymptomatic hypoglycaemia, may be difficult [9].

Continuous glucose monitoring (CGM) allows for continuous measurement of interstitial glucose levels in subcutaneous tissues and evaluation of the detailed blood glucose profile of the patient. CGM includes retrospective CGM (r-CGM), which is used for retrospective examination of lifestyle problems and pharmacotherapy adjustment after understanding the blood glucose profile over several days, and real-time CGM (RT-CGM), which confirms the blood glucose profile in real-time. Studies have shown that utilization of such CGM approaches promotes favourable blood glucose control by changing patient behaviours or pharmacotherapy adjustment [10, 11].

A 2013 meta-analysis that examined the influence of CGM on blood glucose levels in patients with type 2 diabetes mellitus indicated significant improvements in HbA1c levels [12]. However, the aforementioned study included only a few randomized controlled trials (RCTs) and did not examine whether CGM intervention had a direct hypoglycaemic reduction effect or an influence on weight. In the present study, therefore, we aimed to investigate the effects of CGM on blood glucose levels, body weight, blood pressure, and hypoglycaemia in patients with type 2 diabetes mellitus using a meta-analysis of RCTs.

## 2. Materials and Methods

### 2.1. Study Selection

A literature search was performed on 1st February 2018 using MEDLINE (from 1960), Cochrane Controlled Trials Registry (from 1960), and ClinicalTrials.gov. The search strategy was “(type 2 diabet^∗^ or T2DM or NIDDM or non-insulin dependent diabet^∗^) AND [continuous glucose and (monitor^∗^ or sensing or sensor^∗^)] or [continuous subcutaneous glucose and (monitor^∗^ or sensing or sensor^∗^)] or CGM or CGMS or real-time CGM or RT-CGM or flash glucose monitor^∗^ or FGM or sensor-augmented insulin pump or SAP AND (randomized controlled trial or controlled clinical trial or randomized or randomised or placebo or randomly).” The present study included RCTs that evaluated the effect of CGM on blood glucose levels, body weight, hypoglycaemic frequency, and other parameters in type 2 diabetes. Moreover, we included RCTs that compared CGM and SMBG regardless of diet/exercise therapy, oral hypoglycaemic agent use, and injectable formulation administration. The exclusion criteria were as follows: non-RCT studies, those involving animal experiments, those that targeted patients with gestational diabetes, those with insufficient data for analysis, and duplicate literature. Two authors (SI and RK) independently assessed whether each document satisfied the eligibility criteria established herein. In case of disagreements between interpretations by the two authors, a third reviewer (KM) was consulted.

### 2.2. Data Extraction and Quality Assessment

We created a data extraction form listing the characteristics of studies included in the present study (i.e., key author's name, publication year, study location, sample size, patient's baseline information, basic treatment, and treatment duration). Continuous variables were expressed as mean values, standard deviations, standard errors, or 95% confidence intervals (CIs), whereas binary variables were expressed as percentages (%). Studies comparing one SMBG group with two or more intervention groups were treated as two or more studies sharing an SMBG group. Two authors (SI and RK) independently evaluated the quality of research included in the present study. Accordingly, Cochrane's risk of bias tool was used for evaluating quality [12]. Six domains (random sequence generation, allocation concealment, blinding of personnel and participants, blinding of outcome assessors, incomplete data, and selective reporting) were evaluated using low, moderate, and high risk of bias.

### 2.3. Statistical Analysis

Given that continuous variables in each study appeared to be expressed using different units, analysis was performed using standardized mean difference (SMD) and 95% CIs. Binary variables were analyzed using the risk ratio (RR) and 95% CIs. When only the standard error or *p* values were described, standard deviation was calculated with reference to the method used by Altman and Bland [13]. When no description for the standard deviation was present, it was calculated from 95% CIs, *t* values, or *p* values [14]. A random effects model was used for analysis; *I*^2^ was used for evaluating statistical heterogeneity (*I*^2^ ≥ 50% was regarded as heterogeneous) [15]. When the number of RCTs included in the analysis was ≥10, a funnel plot was created for evaluating publication bias [14]. Furthermore, previous studies have reported that baseline HbA1c levels and age may affect the influence of CGM on HbA1c levels [16, 17]. Therefore, when heterogeneity was noted, a metaregression analysis was conducted on whether baseline HbA1c levels, age, and frequency of CGM sensor use affected the impact of CGM on HbA1c levels. Moreover, RevMan version 5.3 (Cochrane Collaboration, https://tech.cochrane.org/revman/download, July/2017) and STATA version 12.1 (Stata Corporation LP, College Station, TX) were used for the analysis.

## 3. Results

### 3.1. Description of Included Studies

**Figure 1:**
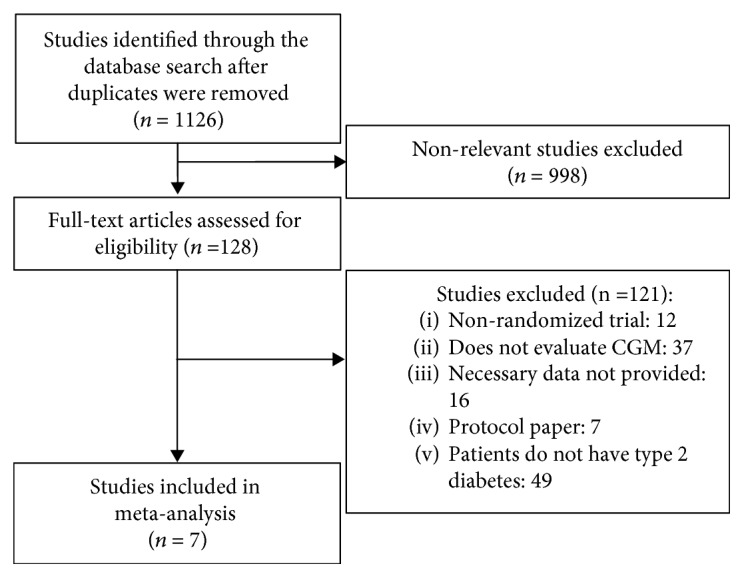
Study flow diagram.

A total of 1126 papers were extracted from the literature search, among which seven RCTs (669 patients) satisfied the eligibility criteria and were included in the meta-analysis (Figure 1) [18–24]. The characteristics of the seven RCTs are summarized in Table 1. Accordingly, three RCTs used RT-CGM [18–20], whereas four used r-CGM [21–24]. The mean age of the subjects was 58.0 years. Moreover, women comprised 39.5% of the subjects, the duration of diabetes was 14.0 years, and the test period was 15.1 weeks.

### 3.2. Assessment of Potential Bias

Among RCTs included herein, proportions of appropriate assessments for each domain were as follows: random sequence generation, 85.7% (6/7); allocation concealment, 85.7% (6/7); blinding of participants and personnel, 0% (0/7); blinding of outcome assessors, 14.2% (1/7); incomplete data, 71.4% (5/7); and selective reporting, 100% (7/7). The quality of the included RCTs varied considerably, with none of the included studies having a low risk of bias. Generally, the overall risk of bias was high, with most of the bias originating from blinding of participants, personnel, and outcome assessors. As there were <10 RCTs, a funnel plot was not created.

### 3.3. HbA1c

**Figure 2:**
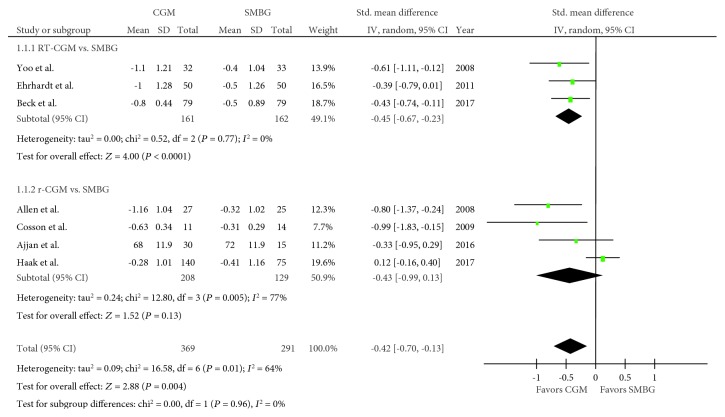
Forest plot presenting the meta-analysis based on standardized mean differences (SMDs) for the effect of CGM versus SMBG on HbA1c levels. SMDs in the individual studies are presented as squares with 95% confidence intervals (CIs) presented as extending lines. Pooled SMD with its 95% CI is presented as a diamond. CGM: continuous glucose monitoring; SMBG: self-monitoring blood glucose; RT-CGM: real-time continuous glucose monitoring; r-CGM: retrospective continuous glucose monitoring.

Seven trials regarding HbA1c were included in the meta-analysis [18–24], with 369 and 291 pooled subjects belonging to the CGM and SMBG groups, respectively. An *I*^2^ value of 64% (*P* = 0.01) confirmed the presence of heterogeneity. The CGM group had significantly lower HbA1c levels than the SMBG group (SMD, −0.42; 95% CI, −0.70–−0.13; *P* = 0.004; Figure 2). When RT-CGM and r-CGM were viewed separately, the comparison between the RT-CGM and SMBG groups resulted in an SMD of −0.45 (95% CI, −0.67–−0.23; *P* < 0.001), whereas the comparison between the r-CGM and SMBG groups resulted in an SMD of −0.43 (95% CI, −0.99–0.13; *P* = 0.13). In addition, despite performing metaregression analysis because of heterogeneity, baseline HbA1c levels (*P* = 0.244) and age (*P* = 0.068) did not affect the impact of CGM on HbA1c.

### 3.4. Body Weight

**Figure 3:**
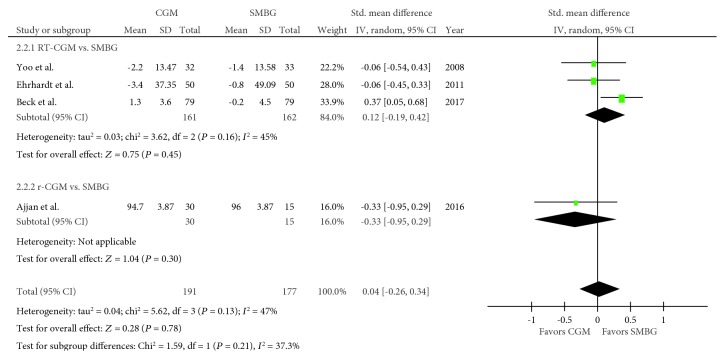
Forest plot presenting the meta-analysis based on standardized mean differences (SMDs) for the effect of CGM versus SMBG on body weight. SMDs in the individual studies are presented as squares with 95% confidence intervals (CIs) presented as extending lines. Pooled SMD with its 95% CI is presented as a diamond. CGM: continuous glucose monitoring; SMBG: self-monitoring blood glucose; RT-CGM: real-time continuous glucose monitoring; r-CGM: retrospective continuous glucose monitoring.

Four trials regarding body weight were included in the meta-analysis [18–20, 23], with 191 and 177 pooled subjects belonging to the CGM and SMBG groups, respectively. An *I*^2^ value of 47% (*P* = 0.13) suggested no heterogeneity. No difference in body weight change was noted between the CGM and SMBG groups (SMD, 0.04; 95% CI, −0.26–0.34; *P* = 0.78; Figure 3). When RT-CGM and r-CGM were viewed separately, the comparison between the RT-CGM and SMBG groups resulted in an SMD of 0.12 (95% CI, −0.19–0.42; *P* = 0.45), whereas the comparison between the r-CGM and SMBG groups resulted in an SMD of −0.33 (95% CI, −0.95–0.29; *P* = 0.30).

### 3.5. Time Spent with Hypoglycaemia (<70 mg/dL) and Hyperglycaemia (>180 mg/dL)

**Figure 4:**
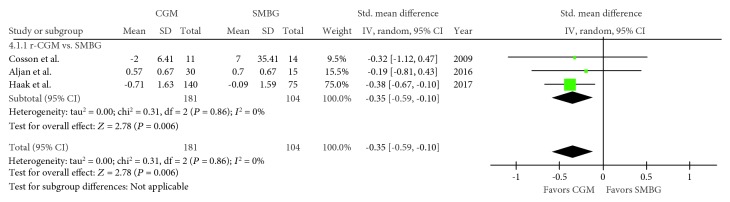
Forest plot presenting the meta-analysis based on standardized mean differences (SMDs) for the effect of CGM versus SMBG on time spent with hypoglycaemia (<70 mg/dL). SMDs in the individual studies are presented as squares with 95% confidence intervals (CIs) presented as extending lines. Pooled SMD with its 95% CI is presented as a diamond. CGM: continuous glucose monitoring; SMBG: self-monitoring blood glucose; RT-CGM: real-time continuous glucose monitoring; r-CGM: retrospective continuous glucose monitoring.

**Figure 5:**
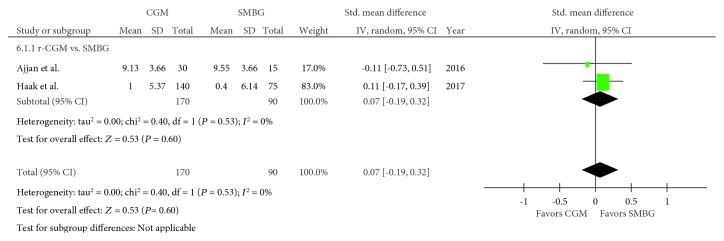
Forest plot presenting the meta-analysis based on standardized mean differences (SMDs) for the effect of CGM versus SMBG on time spent with hyperglycaemia (>180 mg/dL). SMDs in the individual studies are presented as squares with 95% confidence intervals (CIs) presented as extending lines. Pooled SMD with its 95% CI is presented as a diamond. CGM: continuous glucose monitoring; SMBG: self-monitoring blood glucose; RT-CGM: real-time continuous glucose monitoring; r-CGM: retrospective continuous glucose monitoring.

Three trials regarding time spent with hypoglycaemia were included in the meta-analysis [21, 22, 24], with 181 and 104 pooled subjects in the CGM and SMBG groups, respectively. An *I*^2^ value of 0% (*P* = 0.86) suggested no heterogeneity. The CGM group exhibited significantly shorter time spent with hypoglycaemia than the SMBG group (SMD, −0.35; 95% CI, −0.59–−0.10; *P* = 0.006; Figure 4). Moreover, two trials regarding time spent with hyperglycaemia were included in the meta-analysis [21, 24], with 170 and 90 pooled subjects in the CGM and SMBG groups, respectively. An *I*^2^ value of 0% (*P* = 0.53) indicated no heterogeneity. No difference in time spent with hyperglycaemia was observed between the CGM and SMBG groups (SMD, 0.07; 95% CI, −0.19–0.32; *P* = 0.60; Figure 5). Moreover, tests comparing the RT-CGM and SMBG groups were not included in these analyses.

### 3.6. Blood Pressure

**Figure 6:**
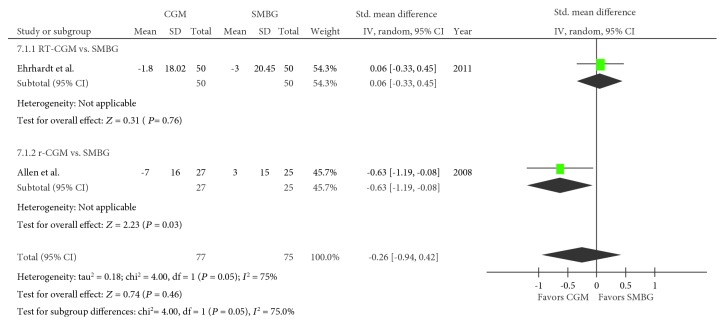
Forest plot presenting the meta-analysis based on standardized mean differences (SMDs) for the effect of CGM versus SMBG on systolic blood pressure. SMDs in the individual studies are presented as squares with 95% confidence intervals (CIs) presented as extending lines. Pooled SMD with its 95% CI is presented as a diamond. CGM: continuous glucose monitoring; SMBG: self-monitoring blood glucose; RT-CGM: real-time continuous glucose monitoring; r-CGM: retrospective continuous glucose monitoring.

**Figure 7:**
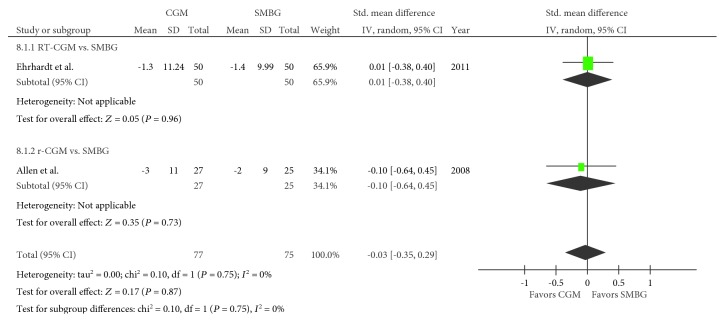
Forest plot presenting the meta-analysis based on standardized mean differences (SMDs) for the effect of CGM versus SMBG on diastolic blood pressure. SMDs in the individual studies are presented as squares with 95% confidence intervals (CIs) presented as extending lines. Pooled SMD with its 95% CI is presented as a diamond. CGM: continuous glucose monitoring; SMBG: self-monitoring blood glucose; RT-CGM: real-time continuous glucose monitoring; r-CGM: retrospective continuous glucose monitoring.

Two trials regarding systolic blood pressure were included in the meta-analysis [19, 21], with 77 and 75 pooled subjects in the CGM and SMBG groups, respectively. An *I*^2^ value of 75% (*P* = 0.05) confirmed heterogeneity. No difference in the systolic blood pressure was observed between the CGM and SMBG groups (SMD, −0.26; 95% CI, −0.94–0.42; *P* = 0.46; Figure 6). When RT-CGM and r-CGM were viewed separately, the comparison between the RT-CGM and SMBG groups resulted in an SMD of 0.06 (95% CI, −0.33–0.45; *P* = 0.76), whereas a comparison between the r-CGM and control or SMBG group resulted in an SMD of −0.63 (95% CI, −1.19–−0.08; *P* = 0.03). The same two trials were used for studying the diastolic blood pressure in the meta-analysis [19, 21]. No difference in the diastolic blood pressure was observed between the CGM and SMBG groups (SMD, −0.03; 95% CI, −0.35–0.29; *P* = 0.87; Figure 7). When RT-CGM and r-CGM were viewed separately, the comparison between the RT-CGM and SMBG groups resulted in an SMD of 0.01 (95% CI, −0.38–0.40; *P* = 0.96), whereas a comparison between the r-CGM and SMBG groups resulted in an SMD of −0.10 (95% CI, −0.64–0.45; *P* = 0.730).

### 3.7. CGM Satisfaction and Quality of Life

Diabetes-specific scales used in the included trials were the Diabetes Treatment Satisfaction Questionnaire (DTSQ), Diabetes Quality of Life (DQoL), Diabetes Distress Scale (DDS), CGM Satisfaction Scale, etc. (Table 2). Accordingly, although three trials [20, 23, 24] evaluated the aforementioned scales, a meta-analysis was not performed because of the different scales used for each study. Two trials utilizing the DTSQ, DQoL, and CGM Satisfaction Scale revealed that treatment satisfaction was higher in the CGM group than in the SMBG group [20, 24]. However, in the remaining trial utilizing the DTSQ [23], no difference in the degree of treatment satisfaction was observed between the CGM and SMBG groups. Two trials utilizing DDS found no significant differences in scores between the CGM and SMBG groups [20].

## 4. Discussion

In this study, we examined the influence of CGM on blood glucose levels, weight, blood pressure, and frequency of hypoglycaemia in patients with type 2 diabetes mellitus using a meta-analysis of RCTs. Accordingly, our results revealed that HbA1c levels and time spent with hypoglycaemia were significantly lower in the CGM group than in the SMBG group. Conversely, no difference in body weight and blood pressure was observed between the CGM and SMBG groups.

One 2013 meta-analysis involving four RCTs that collectively examined the effects of RT-CGM and r-CGM in patients with type 2 diabetes mellitus indicated that the CGM treatment group had significantly lower HbA1c levels than the SMBG group [11]. Similarly, the present study revealed that the CGM group had significantly lower HbA1c levels than the SMBG group. However, when RT-CGM and r-CGM were viewed separately, we found that although the RT-CGM group had predominantly lower HbA1c levels than the SMBG group, no significant difference in HbA1c levels had been found between the r-CGM and SMBG groups. According to a systematic review of patients with type 1 diabetes, RT-CGM has a greater blood glucose-ameliorating effect than r-CGM [25]. The use of RT-CGM helps patients not only adjust diabetes medication dosage but also understand changes in blood glucose levels on a monitor and be conscious of lifestyle factors, such as meals and exercise, thereby ameliorating blood glucose levels [18, 21, 26]. Conversely, r-CGM increases physical activity and blood glucose amelioration and inhibits the onset of complications [21]. Nevertheless, further studies are needed to determine whether RT-CGM improves HbA1c in patients with type 2 diabetes mellitus to a greater extent than r-CGM.

We showed no difference in body weight change between the CGM and SMBG groups. However, although the study by Beck et al. [20] showed that the RT-CGM group tended to have greater body weight than the SMBG group, the other three trials [18, 19, 23] showed no change or even a decrease in body weight. The daily amount of insulin administered in Beck et al.'s study increased compared with the baseline. However, this remained unchanged or decreased in the other three trials. Moreover, Beck et al.'s study revealed that although patients in the RT-CGM group had improved blood glucose levels because of an increase in snacking as a result of hypoglycaemia or an increase in insulin levels to correct blood glucose levels, an increase in body weight could have been present. Accordingly, blood glucose management using CGM in patients with type 2 diabetes mellitus necessitates paying close attention to the insulin dose and changes in weight [26].

With regard to influence on hypoglycaemia, we showed that the RT-CGM group spent less time with hypoglycaemia than the SMBG group. A previous study examining the utility of CGM for type 1 diabetes observed a shortening in the time spent with hypoglycaemia because of CGM intervention. In general, CGM intervention exhibits greater hypoglycaemic effect among patients with high hypoglycaemic frequency at baseline, such as those with type 1 diabetes [17]. Among the studies included in the present meta-analysis, the time spent with hypoglycaemia per day at patient baseline ranged from 3 to 60 min, which may be considered relatively short [22–24]. Nevertheless, CGM intervention shortened the time spent with hypoglycaemia, suggesting its practicality for shortening time spent with hypoglycaemia in patients with type 2 diabetes mellitus. However, given that RCTs comparing the RT-CGM and SMBG groups had not been included in the present analysis, further investigation is necessary.

One study on the effect on blood pressure included herein showed that the CGM group had no reduction in systolic and diastolic blood pressure compared with the SMBG group. In another study included herein, Allen et al. found that the r-CGM group exhibited lower blood pressure during the collection period than the SMBG group. However, as indicated in a previous study [11], given the inclusion of counselling on exercise therapy based on r-CGM data, the independent impact of r-CGM might have not been observed. However, most of the patients in trials included herein had been administered hypotensive medication for blood pressure management. Accordingly, baseline blood pressure management appeared to be the reason why intervention effects of CGM had not been observed. Moreover, assessing the influence of CGM on blood pressure had been generally difficult given the few studies included.

Although a meta-analysis regarding treatment satisfaction after CGM intervention had not been conducted, the present study included one trial [20, 24] that indicated increased treatment satisfaction and another [23] in which no change was noted. Accordingly, the shortening of time spent with hypoglycaemia has been speculated to be the reason for such differences. In a previous study on patients with type 1 diabetes, the decrease in hypoglycaemic frequency had been indicated to be closely related to patient satisfaction [27]. In our study, there are similar observations wherein a shortening of time spent with hypoglycaemia because of CGM in two trials resulted in increased treatment satisfaction, but limited shortening of time spent with hypoglycaemia in one study resulted in unchanged satisfaction. Hence, based on the trials involving patients with type 2 diabetes mellitus included herein, the shortening of time spent with hypoglycaemia because of CGM intervention may perhaps lead to increased treatment satisfaction.

Large-scale clinical trials have shown that strict blood glucose management contributes to the reduction of the risk for vascular complications in patients with type 2 diabetes mellitus [3, 4]. However, avoiding the risk of hypoglycaemia and maintaining patient QOL are also extremely important for glucose management. The present meta-analysis showed that the CGM group exhibited a significantly greater degree of HbA1c reduction (a decrease of approximately 1% from the baseline value) and shorter time spent with hypoglycaemia than the SMBG group. A ≥0.5% improvement in HbA1c levels or a ≥10% improvement from baseline values contributes to the inhibition of future cardiovascular events and has been indicated as clinically significant amelioration [28–30]. Given that hypoglycaemia and blood glucose fluctuations, which are believed to be related to various poor outcomes, could be underestimated in patients in type 2 diabetes mellitus [31], understanding detailed blood glucose profiles through CGM may be useful. In recent years, the increase in healthcare costs has been noted as a problem. Reportedly, CGM intervention is useful in terms of cost effectiveness in patients with type 1 diabetes [32] and in those with type 2 diabetes [33, 34], although the number of reports is limited for the latter type of patients. Further investigations are needed on effects of CGM intervention in patients with type 2 diabetes to alleviate complications, to reduce the incidence of cardiovascular disease, and to improve QOL and cost effectiveness.

The present study had several limitations. First, given the few number of RCTs included, the present study might have had insufficient power to detect differences between groups. Second, although previous studies on RT-CGM interventions had indicated that the frequency of CGM sensor use influences its effects on HbA1c levels [35], this had not been examined because of a lack of sufficient data. Third, we cannot deny the possibility that some literature could have been missed while searching the databases, which could have influenced the results of the present study. Fourth, the observation period and evaluation items of each RCT included herein varied greatly. Therefore, it appeared necessary to pay close attention to the interpretation of the results and generalization. Finally, the quality of RCTs included in the present study was generally low. Moreover, given the presence of heterogeneity, there could be concern regarding the validity of the results derived from the present study.

The present study examined the effects of CGM on blood glucose levels, body weight, blood pressure, and hypoglycaemia in patients with type 2 diabetes mellitus using a meta-analysis of RCTs. The results revealed that the CGM group had significantly lower HbA1c levels and shorter time spent with hypoglycaemia than the SMBG group. On the other hand, no difference in body weight and blood pressure had been observed between the CGM and SMBG groups. As previously mentioned, given the few RCTs included as well as the presence of heterogeneity, care may be needed when interpreting the results of the present study. Accordingly, further studies addressing the limitations presented herein may be necessary.

## Figures and Tables

**Table 1:** Characteristics of CGM interventions included in the present meta-analysis compared with SMBG interventions.

Reference	Year	Region	No. of patients	Age (years)	% women	BMI (kg/m^2^)	Body weight (kg)	Duration of DM (years)	HbA1c (%)	Hypertension (%)	Dyslipidaemia (%)	Prior CVD (%)	Diabetes treatment	Study duration (weeks)	Type of RT-CGM	Frequency of sensor usage (%)
RT-CGM vs. SMBG																
Yoo et al. [18]	2008	Korea	65	57.5	50	25.7	65.7	13.3	8.7	NR	NR	NR	Insulin alone or OADs alone or insulin+OADs	12	Guardian-RT MiniMed (Medtronic)	NR
Ehrhardt et al. [19]	2011	US	100	60	56	32.7	197	NR	8.2	NR	NR	NR	Diet+exercise or OADs alone or OADs+GLP-1 or basal insulin, alone or in combination	12	Dexcom SEVEN (Dexcom)	68
Beck et al. [20]	2017	US	158	60	43.9	37	105	18	8.5	82	63	4	Insulin alone or insulin+OADs	24	Dexcom G4 Platinum (Dexcom)	92
r-CGM vs. SMBG																
Allen et al. [21]	2008	US	52	57	48	33.8	NR	8.5	8.4	NR	NR	NR	Diet+exercise	8	Medtronic MiniMed (Medtronic)	NR
Cosson et al. [22]	2009	France	25	57	27.2	30	NR	10.5	9.2	NR	NR	NR	Insulin alone or insulin+OADs	12	The GlucoDay system (Menarini Diagnostics)	NR
Ajjan et al. [23]	2016	UK	45	55.5	26.7	33.2	93.9	15.8	9.2	NR	NR	NR	Insulin	100 (days)	FreeStyle Navigator (Abbotts)	NR
Haak et al. [24]	2017	Germany	224	59.5	25	33.3	99	18	7.5	NR	NR	NR	Insulin or CSII	24	FreeStyle Libre Pro (Abbotts)	NR

Unless otherwise indicated, data are shown as mean values. CGM: continuous glucose monitoring; SMBG: self-monitoring blood glucose; RT-CGM: real-time continuous glucose monitoring; r-CGM: retrospective continuous glucose monitoring; DM: diabetes mellitus; BMI: body mass index; CVD: cardiovascular diseases; OADs: oral antidiabetic drugs; CSII: continuous subcutaneous insulin infusion; NR: not reported.

**Table 2:** Changes in various patient-reported outcome scores in the CGM and SMBG groups.

	Within-group change, mean (SD)				Between-group change, mean (SD)		
	CGM group		SMBG group		CGM group	SMBG group	*P* value^∗^
	Baseline	End of study	Baseline	End of study			
DTSQ							
Ajjan et al. [23]	—	13.39	—	13.52	—	—	0.936
Haak et al. [24]	—	13.1 (0.5)	—	9.0 (0.7)	—	—	<0.001^∗^
DQoL							
Haak et al. [24]	—	—	—	—	−0.2 (0.0)	0.0 (0.0)	0.025^∗^
DDS							
Beck et al. [20]	1.9 (0.8)	1.8 (0.9)	2.0 (0.8)	1.8 (0.6)	—	—	—
CGM Satisfaction Scale							
Beck et al. [20]	—	4.3 (0.4)	—	—	—	—	—

CGM: continuous glucose monitoring; DTSQ: Diabetes Treatment Satisfaction Questionnaire; DQoL: Diabetes Quality of Life; DDS: Diabetes Distress Scale. ^∗^*P* < 0.05.
